# Correction: Retinal motion statistics during natural locomotion

**DOI:** 10.7554/eLife.91492

**Published:** 2023-08-01

**Authors:** Karl S Muller, Jonathan Matthis, Kathryn Bonnen, Lawrence K Cormack, Alex C Huk, Mary Hayhoe

**Keywords:** Human

Muller KS, Matthis J, Bonnen K, Cormack LK, Huk AC, Hayhoe M. 2023. Retinal motion statistics during natural locomotion. *eLife* **12**:e82410. doi: 10.7554/eLife.82410.

Published 3 May 2023

After publication we were alerted to an error in the y-axis of some of the figures. The affected figures are Figure 4b, 5b, 6b, 7b, 15 (2nd column), 16 (2nd column). There was an error in how the tick marks on the y axis were generated. In the original figures, the axis ticks on the lower axis ticks appeared unequal because of rounding error of tick steps. Note that the upper part of the y axis is intentionally a different scale from the lower part.

The error has been corrected across all of figures where the speed distributions appear. The corrected Figures 4, 5, 6, 7, 15 and 16 are shown in order below.

The corrected Figure 4 is shown here:

**Figure 1:**
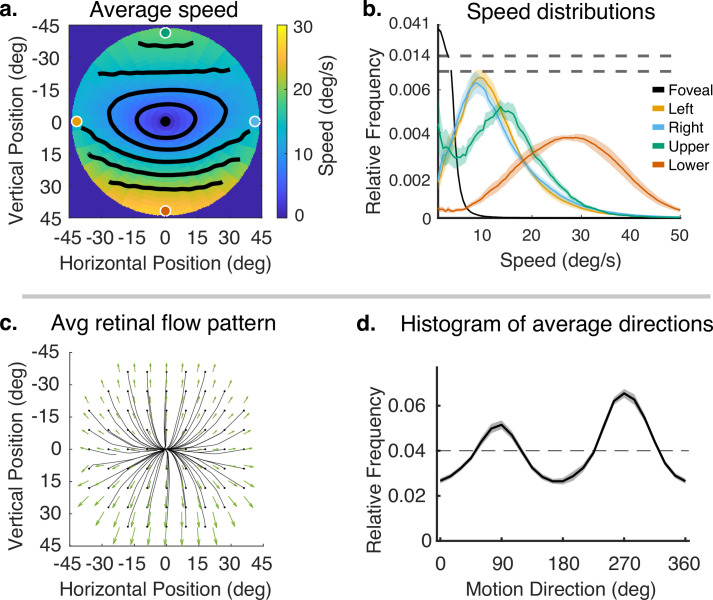


The originally published Figure 4 is shown here for reference:

**Figure 2:**
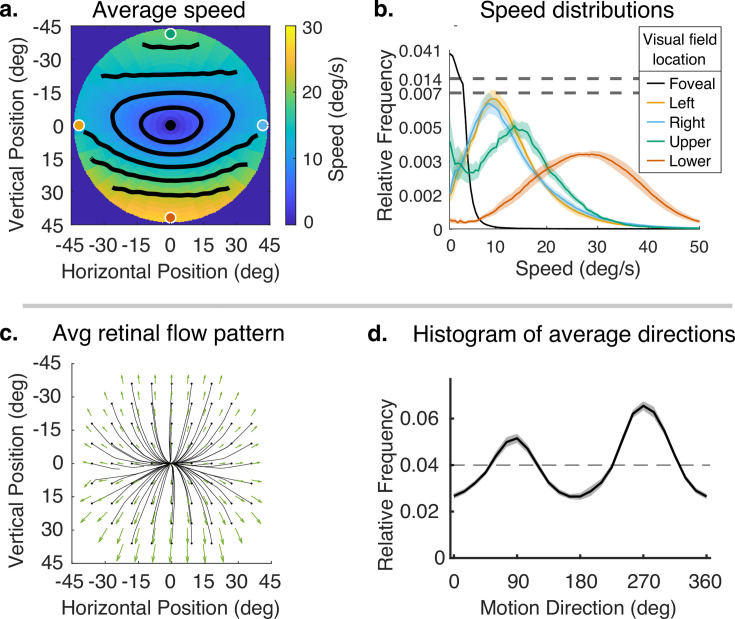


The corrected Figure 5 is shown here:

**Figure 3:**
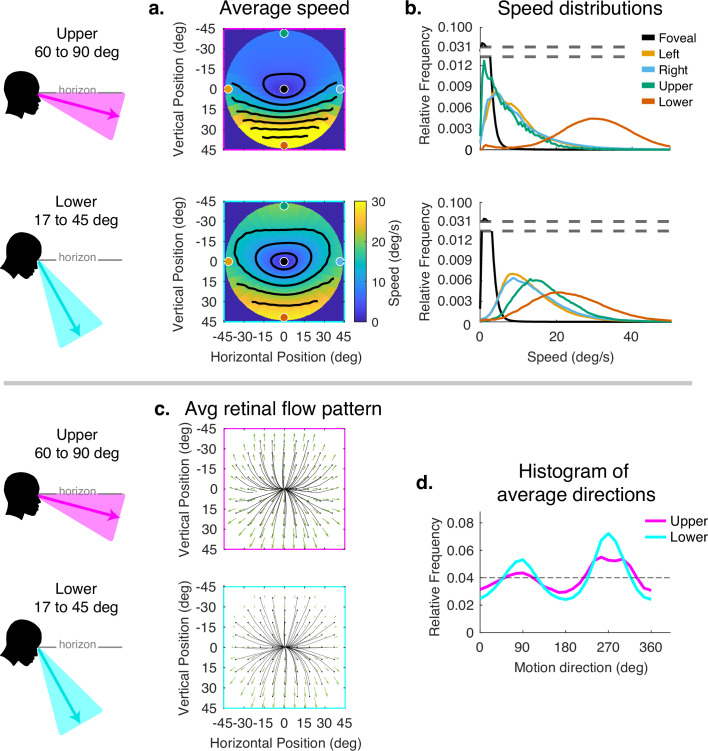


The originally published Figure 5 is shown here for reference:

**Figure 4:**
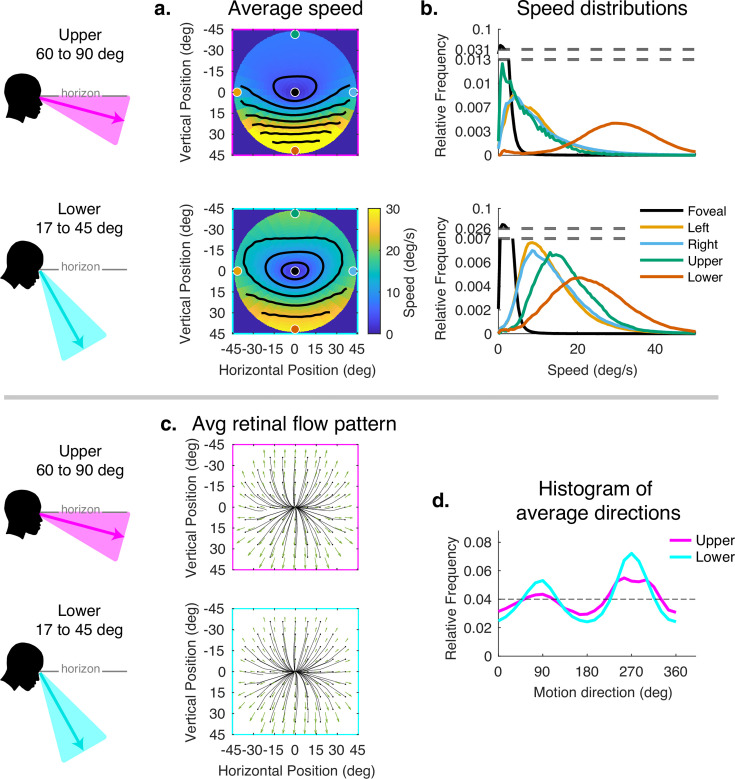


The corrected Figure 6 is shown here:

**Figure 5:**
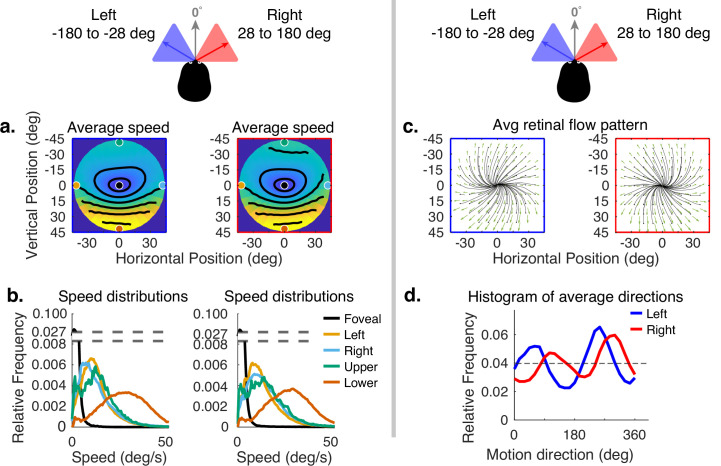


The originally published Figure 6 is shown here for reference:

**Figure 6:**
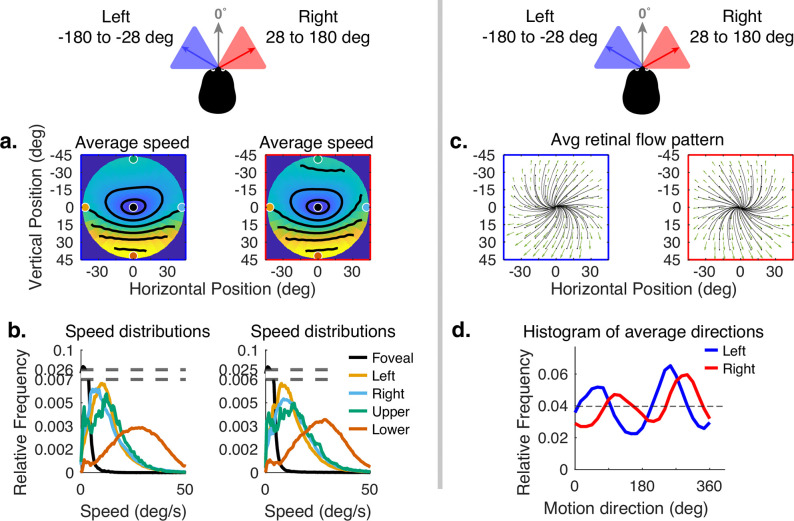


The corrected Figure 7 is shown here:

**Figure 7:**
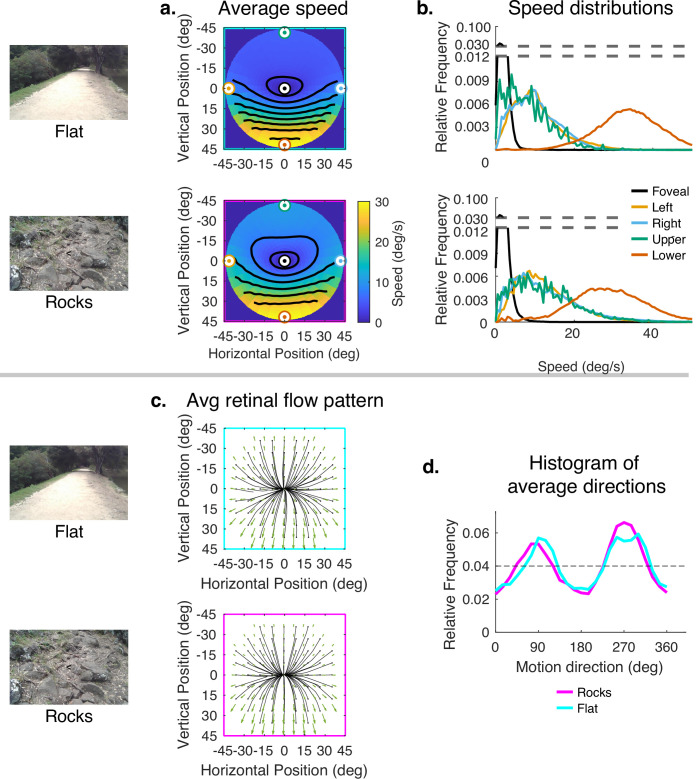


The originally published Figure 7 is shown here for reference:

**Figure 8:**
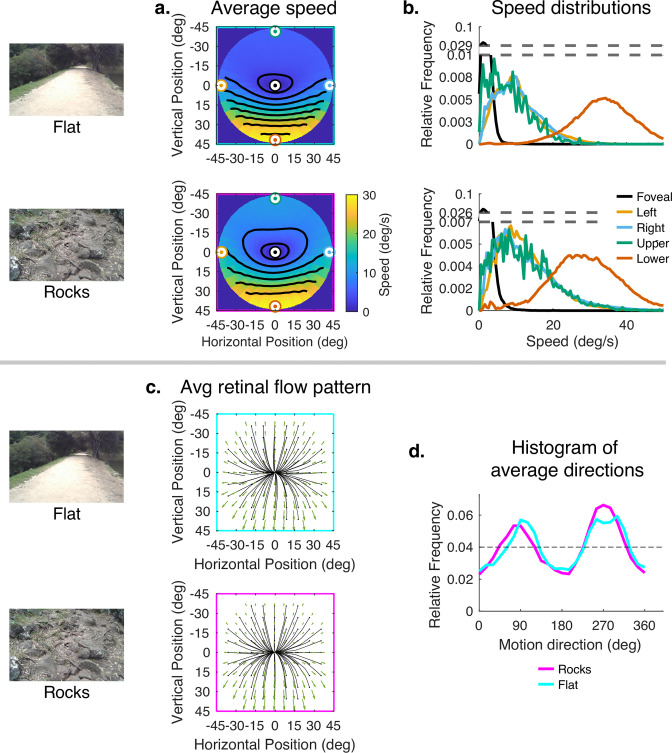


The corrected Figure 15 is shown here:

**Figure 9:**
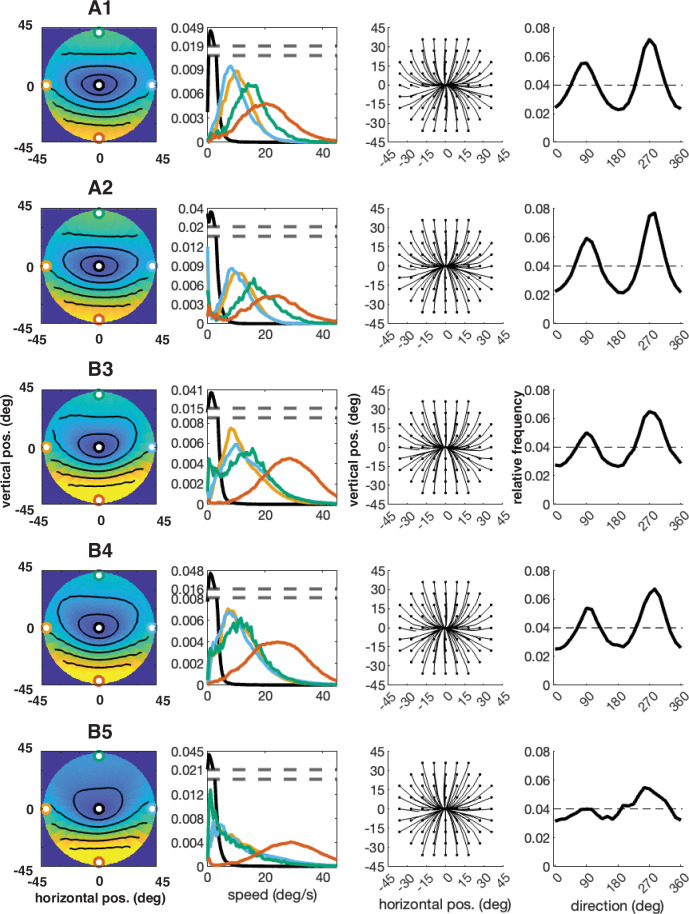


The original Figure 15 is shown here for reference:

**Figure 10:**
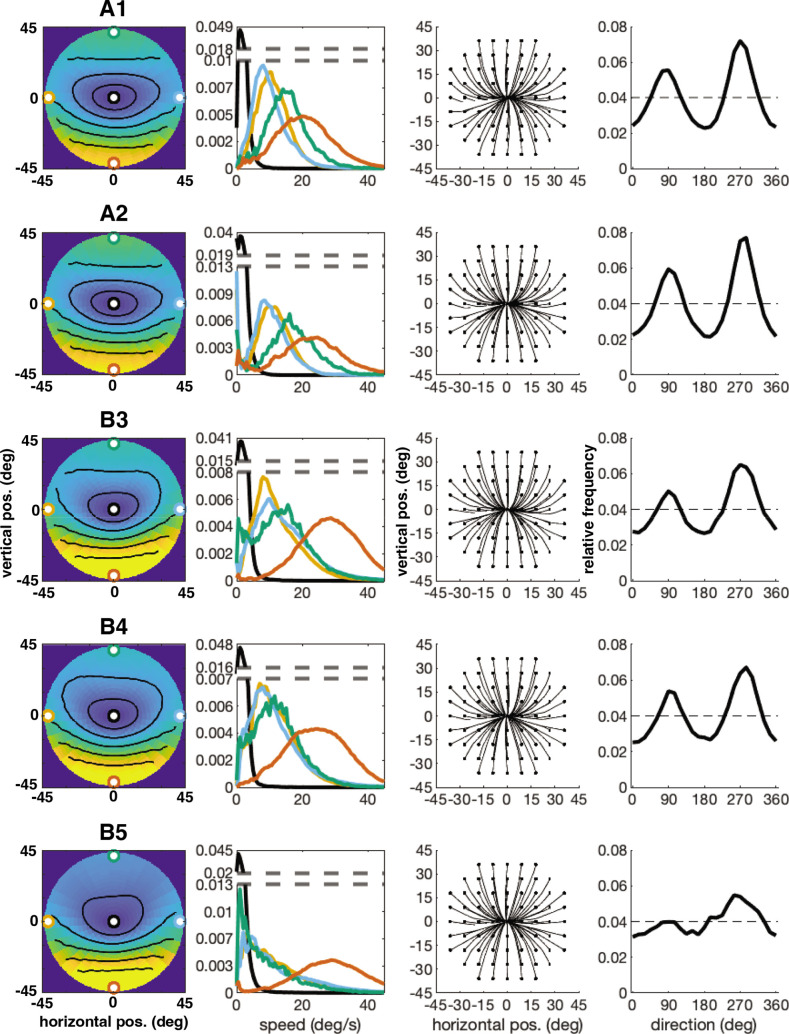


The corrected Figure 16 is shown here:

**Figure 11:**
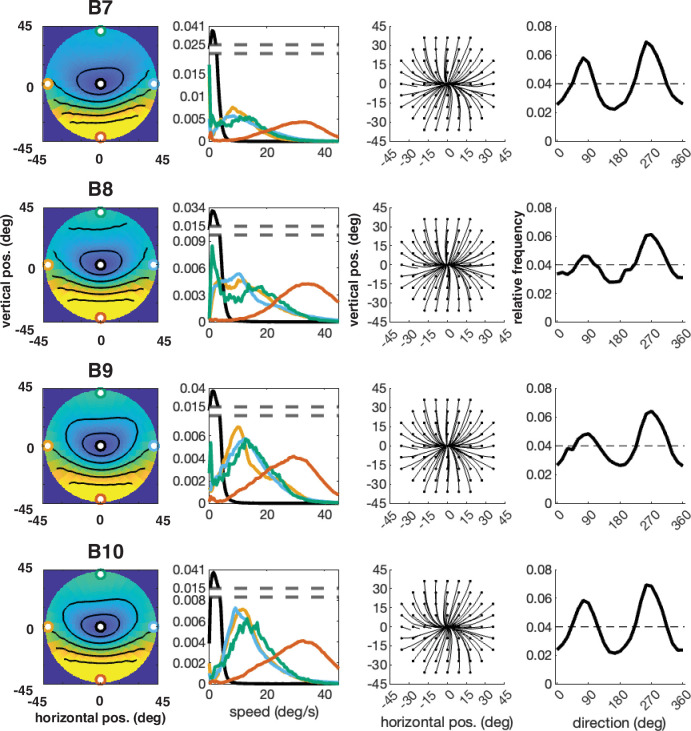


The originally published Figure 16 is shown here for reference:

**Figure 12:**
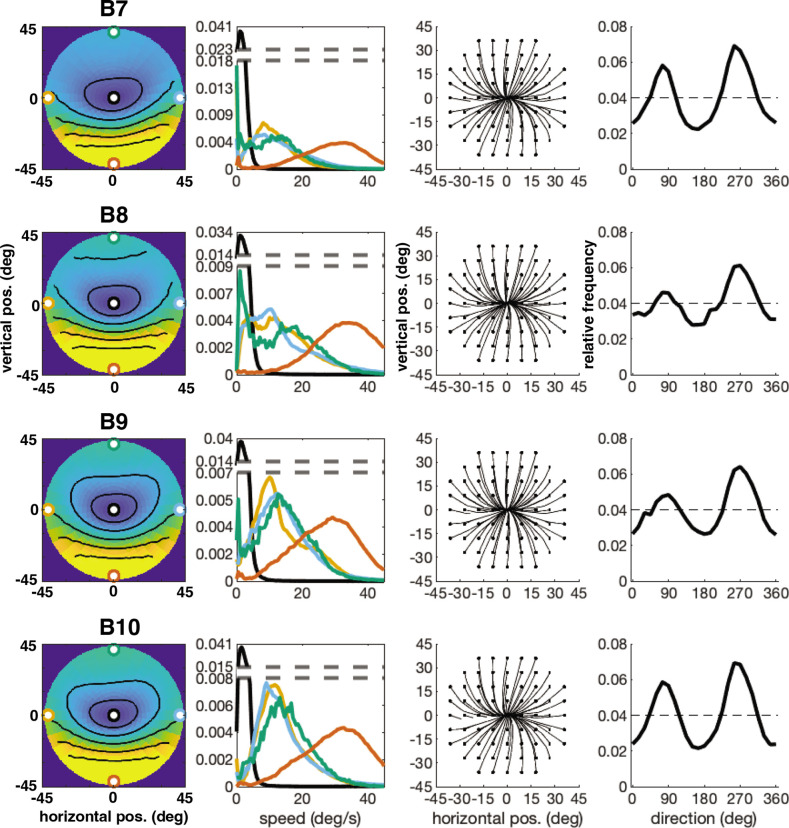


The article has been corrected accordingly.

